# Reporting of health-related quality of life in randomized controlled trials involving palliative systemic therapy for esophagogastric cancer: a systematic review

**DOI:** 10.1007/s10120-018-0792-3

**Published:** 2018-01-29

**Authors:** Emil ter Veer, Jessy Joy van Kleef, Mirjam A. G. Sprangers, Nadia Haj Mohammad, Martijn G. H. van Oijen, Hanneke W. M. van Laarhoven

**Affiliations:** 1 Department of Medical Oncology, Cancer Center Amsterdam, Academic Medical Center, University of Amsterdam, Meibergdreef 9, 1105 AZ Amsterdam, The Netherlands; 2 Department of Medical Psychology, Amsterdam Public Health Research Institute, Academic Medical Center, University of Amsterdam, Meibergdreef 9, 1105 AZ Amsterdam, The Netherlands; 3 Department of Medical Oncology, University Medical Center Utrecht, Heidelberglaan 100, 3584 CX Utrecht, The Netherlands

**Keywords:** Quality of life, Randomized controlled trial, Esophageal cancer, Gastric cancer, Systemic therapy

## Abstract

**Background:**

Health-related quality of life (HRQoL) assessments are increasingly incorporated into oncological randomized controlled trials (RCTs). The quality of HRQoL reporting in RCTs concerning palliative systemic treatment for advanced esophagogastric cancer is currently unknown. Therefore, we conducted a systematic review to investigate the quality of HRQoL reporting over time.

**Methods:**

PubMed, CENTRAL and EMBASE were searched for RCTs concerning systemic treatment for advanced esophagogastric cancer up to February 2017. The Minimum Standard Checklist for Evaluating HRQoL Outcomes in Cancer Clinical Trials was used to rate the quality of HRQoL reporting. Univariate and multivariate generalized linear regression analysis was used to investigate factors affecting the quality of reporting over time.

**Results:**

In total, 37 original RCTs (*N* = 10,887 patients) were included. The quality of reporting was classified as ‘very limited’ in 4 studies (11%), ‘limited’ in 24 studies (65%), and ‘probably robust’ in 9 studies (24%). HRQoL reporting did not improve over time, and it did not improve following the publication of the CONSORT-PRO statement in 2013. The publication of HRQoL findings in a separate article and second-line treatment were associated with better reporting.

**Conclusions:**

HRQoL reporting in RCTs concerning palliative systemic therapy for advanced esophagogastric cancer is limited and has not improved over time. This systematic review provides specific recommendations for authors to improve HRQoL reporting: formulate hypotheses a priori, clearly describe instrument administration, and handle missing data and interpret findings appropriately.

**Electronic supplementary material:**

The online version of this article (10.1007/s10120-018-0792-3) contains supplementary material, which is available to authorized users.

## Introduction

Cancers of the esophagus and stomach are the second and sixth most common causes of cancer-related deaths worldwide [1]. Patients with unresectable locally advanced and metastatic disease can be offered palliative systemic treatment to prolong survival, to offer symptom relief, and to improve or maintain health-related quality of life (HRQoL) [2, 3].

Recognition of the importance of HRQoL is reflected in the fact that HRQoL is assessed in randomized clinical trials (RCTs) with increasing frequency. In the past decades, an increase in HRQoL assessments was shown of 3.6% of trials from all disciplines and 6.7% of cancer trials. However, considerable variation among these HRQoL assessments in the instruments used has been noted, and the reporting of methods and results has often been inadequate [4, 5]. Also, a more recent study has shown that even though more RCT reports are meeting the quality standards for HRQoL assessment, the analysis and reporting of data and the presentation of findings remain highly variable [6]. Inadequate reporting of HRQoL in clinical trials may lead to a loss of valuable information or may even mislead clinical decision-making [7].

In the field of oncological RCTs, Efficace and colleagues showed that the reporting of HRQoL in RCTs of high-incidence diseases (i.e., breast, colorectal, prostate, and lung cancers) has improved over the years [7]. In contrast, in the majority of studies investigating curative treatment for esophageal cancer (a disease with a low incidence), the reporting of HRQoL was limited [8]. The reporting of HRQoL in studies of palliative therapy for advanced esophagogastric cancer has not yet been investigated. Given the limited remaining life span of this patient group, an emphasis on HRQoL is paramount. Therefore, we systematically reviewed the literature to determine the quality of HRQoL reporting in RCTs that involve palliative systemic therapy for patients with esophagogastric cancer. The following research questions were formulated. (1) What is the quality of HRQoL outcome reporting in locally advanced esophagogastric cancer? (2) What aspects of HRQoL reporting require improvement in order to facilitate clinical decision-making? (3) Has the quality of HRQoL reporting improved over time? Given the increasing body of literature regarding patient-reported outcome research, we hypothesized that the reporting of HRQoL in unresectable locally advanced and metastatic esophagogastric cancer had improved over time.

## Methods

The PRISMA statement guided the writing of the manuscript. We focused on items that are relevant to the research questions and excluded irrelevant ones (e.g., items related to potential bias with respect to treatment outcomes).

### Search methods

The study protocol was not registered in advance. The online databases PubMed, EMBASE, and the Cochrane Central Register of Controlled Trials (CENTRAL) as well as meeting abstracts from the American Society of Clinical Oncology (ASCO) and the European Society for Medical Oncology (ESMO) were searched for RCTs on palliative systemic therapy for advanced esophagogastric cancer up to February 2017. Details regarding this search can be found in Online Resource 1 in the Electronic supplementary material (ESM). Prospective registration databases such as clinical trials.gov were not searched, as our aim was to assess published reports. For the same reason, no contact with study authors was sought for additional information. Titles, abstracts, and full texts were screened by EtV and NHM. Disagreements were discussed with JJvK until consensus was reached.

### Study selection

Studies were included that met the following criteria: (1) prospective phase II or III RCT design; (2) unresectable, metastatic, or recurrent esophageal, gastroesophageal junction (GEJ), or gastric cancer; (3) palliative systemic therapy (i.e., chemotherapy and/or targeted therapy); (4) full-text articles published in English; and (5) HRQoL was measured with validated questionnaires. Studies using self-constructed nonvalidated HRQoL questionnaires were not eligible because of their nonreproducible nature or a lack of information regarding their psychometric properties.

### Data extraction

Data extraction was conducted by EtV and JJvK using Microsoft Excel. The following baseline characteristics of the included studies and patients were extracted: number of patients enrolled in the study, gender, age, performance status, tumor histology, tumor location, and disease status. The following characteristics regarding HRQoL reporting were extracted: the presence of a hypothesis a priori, the rationale for the HRQoL instrument, psychometric properties, cultural validity, HRQoL domains, instrument administration, baseline compliance, timing of assessments, documentation of missing data, and the clinical significance and presentation of the results in the discussion section. The quality of HRQoL reporting in each article was rated using the Minimum Standard Checklist for Evaluating HRQoL Outcomes in Cancer Clinical Trials checklist [9]. Articles were rated independently by EtV and JJvK. Discrepancies were discussed until a consensus was reached. The checklist consists of eleven items that can be scored as ‘yes’ (one point) or ‘no’ (zero points), and contains four domains: conceptual, measurement, methodology, and interpretation. Two items (‘a priori hypothesis stated’ and ‘cultural validity verified’) could also be evaluated as ‘not applicable’ (N/A) if the study explicitly stated that the HRQoL assessment was intended for exploratory investigations only or if the HRQoL measure was validated in the same population as that of the trial. When RCTs used validated measures for their study population, all items in the measurement domain (i.e., ‘psychometric properties reported,’ ‘cultural validity verified,’ and ‘adequacy of domains covered’) were scored as ‘yes.’ Three mandatory items of the checklist are: ‘psychometric properties reported,’ ‘baseline compliance reported,’ and ‘reasons for missing data reported.’ The checklist classifies the HRQoL reporting into the following categories: ‘very limited’ (score 0–4), ‘limited’ (score 5–7 or ‘no’ on one or more of the mandatory items), and ‘probably robust’ (score 8–11 and ‘yes’ on all mandatory items). Studies classified as ‘probably robust’ are most likely to have an impact on clinical decision-making [9].

### Statistical analysis

For statistical analysis, the quality of HRQoL reporting—our main outcome—was expressed as an adjusted checklist score (ACS). The ACS was calculated for each study report by dividing the raw item score by the total number of applicable items. Higher ACS scores imply better quality of reporting. Descriptive statistics were used to gain insight into the quality of reporting. To assess the extent to which the quality of HRQoL reporting has improved over time, the variance and change in the ACS over time was graphically assessed using a scatterplot. Subsequently, a univariate generalized linear regression analysis with a binomial distribution, a logit link function, and robust standard errors was performed. Herewith, the independent variable ‘time’ is expressed as the year of publication and the dependent variable ‘quality of HRQoL reporting’ is expressed as the ACS. Predicted values in our model can range between 0 and 1. In order to investigate other associations of study characteristics with the quality of HRQoL reporting, the following covariates were considered in the regression analysis: (1) whether or not the study reported statistically significant differences in HRQoL results at any scale between arms or within arms over time (no vs yes); (2) if a separate article with HRQoL results was published (no vs yes); (3) the presence of an appendix or supplementary data (no vs yes); (4) intention-to-treat sample size (continuous); (5) type of endpoint (primary vs secondary); and (6) type of therapy line (first vs second vs third). Furthermore, we investigated if there were differences in the median ACS between studies that were published before versus after the publication of the CONSORT-PRO statement [10] using the Mann–Whitney *U* test. Statistical significance was reached at the 5% level and all analyses were performed using STATA version 14 for Windows.

## Results

### Literature search

**Fig. 1:**
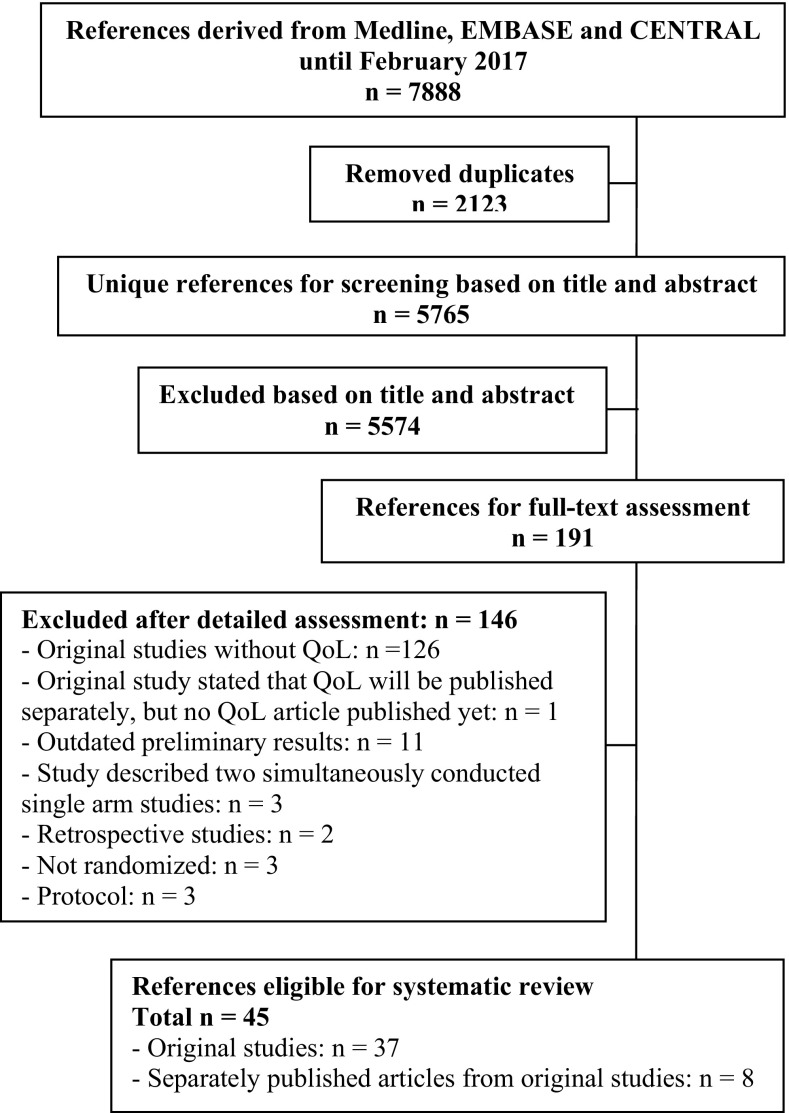
Flowchart of the studies included

One hundred sixty-four RCTs investigating palliative systemic therapy for advanced esophagogastric cancer were eligible. Among these, 37 unique RCTs (*N* = 10,887 patients) reported on HRQoL [11–56]. More details regarding the number of studies screened, assessed for eligibility, and included in the review can be found in Fig. 1. Eight studies (21.6%) published HRQoL findings separately. The year of publication of the studies ranged from 1997 to 2017. Major baseline characteristics of the included studies are shown in Table 1.

**Table 1:** Baseline characteristics

Original study–substudy	*N*	Arms	Male *N* (%)	Median age (range)	Metastasized *N* (%)	WHO ≥ 2 *N* (%)	Esophagus *N* (%)	GEJ *N* (%)	Stomach *N* (%)	HRQoL measure	ACS
First-line treatment											
Ajani (2010) [11]–Bodoky (2015) [12]	521	Cis + S-1	382 (73)	59 (18–83)	497 (95)	0 (0)	0 (0)	82 (16)	438 (84)	FACT-Ga, CCSQ	0.91
	508	Cis + 5-FU	347 (68)	60 (20–85)	488 (96)	0 (0)	0 (0)	88 (17)	417 (82)		
Al-Batran (2013) [13]–Kripp (2014) [14]	72	DTX + Ox +5-FU/Lv	51 (71)	69 (max: 81)	50 (69)	5 (7)	0 (0)	27 (37)	45 (63)	EORTC QLQ-C30, STO22	0.73
	71	Ox + 5-FU/Lv	45 (63)	70 (max: 82)	49 (68)	6 (8)	0 (0)	24 (34)	47 (66)		
Bouché (2004) [15]	45	5-FU/Lv	37 (82)	64 (45–75)	45 (100)	12 (27)	0 (0)	13 (29)	32 (71)	EORTC QLQ-C30	0.64
	44	Cis + 5-FU/Lv	35 (80)	64 (43–76)	44 (100)	22 (25)	0 (0)	13 (30)	31 (70)		
	45	Iri + 5-FU/Lv	38 (84)	65 (37–76)	45 (100)	10 (22)	0 (0)	15 (33)	30 (67)		
Bramhall (2002) [16]	185	Marimastat	131 (71)	68	136 (74)	0 (0)	0 (0)	0 (0)	185 (100)	EORTC QLQ-C30	0.36
	184	PLB	131 (71)	68	132 (72)	0 (0)	0 (0)	0 (0)	184 (100)		
Dank (2008) [18]–Curran (2009) [17]	170	Iri + 5-FU/Lv	125 (73)	58 (29–76)	163 (97)	1 (1)	0 (0)	34 (20)	136 (80)	EORTC QLQ-C30, EQ5D	0.73
	163	Cis + 5-FU	108 (66)	59 (28–77)	155 (95)	2 (1)	0 (0)	31 (19)	132 (81)		
Duffour (2006) [19]	113	Cis + 5-FU	90 (80)	61 (38–75)	113 (100)	0	9 (8)	10 (9)	45 (40)	SQLI	0.55
	113	5-FU/Lv	83 (74)	62 (41–76)	113 (100)	1 (1)	10 (9)	9 (8)	46 (41)		
Glimelius (1997) [20]	31	Etoposide + 5-FU/Lv	23 (74)	64 (45–75)	NA	*79 (50–100)	0 (0)	9 (29)	22 (71)	EORTC QLQ-C30	0.64
	30	BSC	22 (73)	63 (40–74)	NA	*87 (50–100)	0 (0)	7 (23)	23 (77)		
Gubanski (2010) [21]–Gubanski (2014) [22]	39	DTX + 5-FU/Lv	26 (67)	63 (39–79)	18 (45)	38 (3)	0 (0)	0 (0)	39 (100)	EORTC QLQ-C30	0.80
	39	Iri + 5-FU/Lv	34 (87)	64 (42–75)	22 (57)	34 (12)	0 (0)	0 (0)	39 (100)		
Guimbaud (2014) [23]–Nuemi (2015) [24]	209	Epi + Cis + Cap	154 (74)	61 (28–84)	173 (83)	36 (17)	0 (0)	73 (35)	133 (64)	EORTC QLQ-C30, STO22	0.55
	207	Iri + 5-FU/Lv	155 (75)	61 (29–81)	176 (85)	27 (13)	0 (0)	63 (30)	138 (67)		
Hall (2017) [25]	17	Epi + Ox + Cap	13 (76)	74 (64–82)	17 (100)	6 (35)	5 (29)	2 (12)	10 (59)	EORTC QLQ-C30, OG25, EQ 5D	0.55
	19	Ox + Cap	13 (68)	77 (50–85)	17 (89)	5 (26)	11 (58)	1 (5)	5 (26)		
	19	Cap	15 (79)	75 (57–87)	18 (95)	7 (37)	8 (42)	4 (21)	7 (37)		
Hecht (2016) [26]	249	Lapatinib + Ox + Cap	189 (76)	61 (19–86)	237 (95)	21 (8)	12 (5)	23 (9)	214 (86)	EORTC QLQ-C30, OES18, STO22	0.27
	238	Ox + Cap	176 (74)	59 (27-84)	228 (96)	22 (9)	8 (3)	20 (8)	210 (88)		
Hwang (2017) [27]	26	Cap	16 (62)	77 (70–83)	26 (100)	6 (23)	0 (0)	0 (0)	26 (100)	EORTC QLQ-C30	0.55
	24	Ox + Cap	18 (75)	75 (70–84)	24 (100)	4 (17)	0 (0)	0 (0)	24 (100)		
Kim (2012) [28]	65	Ox + S-1	44 (68)	60 (28–77)	47 (72)	0 (0)	0 (0)	0 (0)	65 (100)	EORTC QLQ-C30	0.55
	64	Ox + Cap	45 (70)	61 (20–75)	46 (72)	0 (0)	0 (0)	0 (0)	64 (100)		
Ohtsu (2011) [29]	387	Bevacizumab + Cis + Cap	257 (66)	58 (22–81)	367 (95)	22 (6)	0 (0)	54 (14)	333 (86)	EORTC QLQ-C30, STO22	0.36
	387	Cis + Cap	258 (67)	59 (22–82)	378 (98)	20 (5)	0 (0)	49 (13)	338 (87)		
Park (2006) [30]	38	PTX + 5-FU	19 (50)	53 (36–73)	38 (100)	8 (21)	0 (0)	0 (0)	38 (100)	EORTC QLQ-C30	0.64
	39	DXT + 5-FU	26 (67)	51 (27–74)	39 (100)	6 (15)	0 (0)	0 (0)	39 (100)		
Park (2008) [31]	45	Iri + 5-FU/Lv	30 (65)	55 (26–73)	13 (29)	11 (24)	0 (0)	0 (0)	45 (100)	EORTC QLQ-C30	0.64
	45	PTX + Iri + 5-FU/Lv	30 (67)	51 (29–70)	14 (31)	7 (16)	0 (0)	0 (0)	45 (100)		
Rao (2010) [32]	35	Matuzumab + Epi + Cis + Cap	24 (69)	59 (29–79)	35 (100)	0 (0)	0 (0)	21 (60)	14 (40)	EORTC QLQ-C30, OES18	0.45
	36	Epi + Cis + Cap	27 (75)	64 (36–76)	36 (100)	0 (0)	0 (0)	20 (56)	16 (44)		
Ross (2002) [33]	289	Epi + Cis + 5-FU	218 (75)	58 (28–78)	154 (53)	60 (21)	95 (33)	61 (21)	125 (43)	EORTC QLQ-C30	0.55
	285	MMC + Cis + 5-FU	226 (79)	59 (29–77)	174 (61)	49 (17)	93 (33)	64 (22)	118 (41)		
Roth (2007) [34]	40	Epi + Cis + 5-FU	30 (75)	59 (32–71)	33 (83)	0 (0)	0 (0)	0 (0)	40 (100)	EORTC QLQ-C30	0.64
	38	DTX + Cis	29 (76)	58 (40–70)	31 (82)	0 (0)	0 (0)	0 (0)	38 (100)		
	38	DTX + Cis	29 (76)	58 (40–70)	31 (82)	0 (0)	0 (0)	0 (0)	38 (100)		
Ryu (2015) [35]	306	S-1 + Cis (3 weeks)	231 (75)	60 (27–74)	306 (100)	10 (3)	0 (0)	306 (100)		EQ-5D	0.45
	309	S-1 + Cis (5 weeks)	233 (75)	59 (29–74)	309 (100)	3 (1)	0 (0)	309 (100)			
Sadighi (2006) [36]	42	Epi + Cis + 5-FU	34 (81)	**57.2 (SD 9.83)	12 (29)	NA	0 (0)	0 (0)	42 (100)	EORTC QLQ-C30	0.73
	36	DTX + Cis + 5-FU	31 (70)	**55.4 (SD 14.04)	11 (31)	NA	0 (0)	0 (0)	44 (100)		
Tebbutt (2002) [37]	125	5-FU	96 (76)	72 (52–84)	71 (38)	37 (30)	29 (24)	33 (27)	55 (45)	EORTC QLQ-C30	0.45
	129	5-FU + MMC	95 (75)	72 (52–84)	73 (58)	44 (35)	27 (21)	30 (24)	69 (54)		
Tebbutt (2010) [38]	50	DTX + Cis + 5-FU	42 (84)	61 (35–82)	1 (2)	1 (2)	11 (22)	13 (26)	26 (52)	EORTC QLQ-C30, OES18, STO22	0.55
	56	DTX + Cap	42 (75)	59 (32–79)	2 (4)	2 (4)	20 (36)	13 (23)	23 (41)		
Tebbutt (2016) [39]	37	Pmab + DTX + Cis + Cap/5-FU	33 (87)	64 (40–79)	37 (100)	2 (5)	15 (40)	10 (26)	13 (34)	EORTC QLQ-C30, OES18, STO22	0.55
	39	DTX + Cis + Cap/5-FU	30 (77)	59 (37–77)	39 (100)	3 (8)	13 (33)	11 (28)	15 (39)		
Van Cutsem (2006) [41]–Ajani (2007) [40]	227	DTX + Cis + 5-FU	159 (72)	55 (26–79)	213 (96)	3 (1)	0 (0)	42 (19)	185 (81)	EORTC QLQ-C30, EQ 5D	0.82
	230	Cis + 5-FU	158 (71)	55 (25–76)	217 (97)	3 (1)	0 (0)	56 (25)	174 (25)		
Bang (2010) [55]–Satoh (2014) [56]	298	Tmab + Cis + Cap/5-FU	226 (77)	**59.4 (10.8)	284 (97)	30 (10)	0 (0)	58 (20)	236 (80)	EORTC QLQ-C30, STO22, VAS-PAIN	0.82
	296	Cis + Cap/5-FU	218 (75)	**58.5 (11.2)	282 (97)	27 (9)	0 (0)	48 (17)	242 (83)		
Wadler (2002) [42]	12	5-FU + HU + INF-a-2a + Filgrastim	8 (67)	59 (41–77)	7 (58)	0 (0)	0 (0)	12 (100)		FACT–Fatigue & IMFQ	0.55
	11	Doxo + DTX	7 (64)	58 (43–76)	1 (13)	0 (0)	0 (0)	11 (100)			
Webb (1997) [44]–Waters (1999) [43]	126	Epi + Cis + 5-FU	99 (79)	59 (35–79)	79 (63)	30 (24)	27 (21)	27 (21)	72 (57)	EORTC QLQ-C30	0.64
	130	Doxo + 5-FU + MTX	110 (85)	60 (29–78)	79 (61)	32 (25)	24 (18)	33 (25)	73 (56)		
Yoshino (2016) [45]	146	S-1	107 (73)	74 (32–94)	20 (13)	5 (3)	0 (0)	0 (0)	146 (100)	FACT-BRM	0.55
	149	S-1 + Lentinan	101 (68)	73 (44–93)	23 (15)	8 (5)	0 (0)	0 (0)	149 (100)		
Beyond first-line treatment											
Dutton (2014) [46]	224	Gefitinib	36 (16)	65 (58–70)	NA	50 (22)	0 (0)	51 (24)	168 (75)	EORTC QLQ-C30, OG25	0.90
	225	PLB	189 (84)	65 (58–71)	NA	44 (20)	0 (0)	44 (19)	173 (77)		
Ford (2014) [47]	84	DTX + BSC	69 (82)	65 (29–84)	73 (87)	14 (17)	18 (22)	27 (32)	39 (46)	EORTC QLQ-C30, STO22	0.91
	84	BSC	67 (80)	66 (36–84)	74 (88)	12 (14)	15 (18)	32 (38)	37 (44)		
Fuchs (2014) [48]	238	Ramucirumab	169 (71)	60 (52–67)	NA	0 (0)	0 (0)	60 (25)	178 (75)	EORTC QLQ-C30	0.73
	117	BSC	79 (68)	60 (51–71)	NA	1 (1)	0 (0)	30 (26)	87 (74)		
Lee (2016) [49]	23	DTX	18 (78)	56 (34–68)	22 (96)	0 (0)	0 (0)	0 (0)	23 (100)	EORTC QLQ-C30, STO22	0.45
	23	DTX + Cis	20 (87)	55 (38–74)	18 (78)	2 (9)	0 (0)	0 (0)	23 (100)		
	23	DTX + S-1	14 (61)	55 (39–68)	18 (78)	2 (9)	0 (0)	0 (0)	23 (100)		
Li (2016) [50]	176	Apatinib 850 mg once daily	132 (75)	58 (32–71)	NA	0 (0)	0 (0)	39 (22)	122 (69)	EORTC QLQ-C30	0.45
	91	PLB + BSC	69 (76)	58 (28–70)	NA	0 (0)	0 (0)	21 (23)	65 (72)		
Li (2013) [51]	47	Apatinib 850 mg once daily	39 (83)	55	43 (91)	0 (0)	0 (0)	47 (100)		EORTC QLQ-C30	0.36
	46	Apatinib 425 mg twice daily	34 (74)	53	45 (98)	0 (0)	0 (0)	46 (100)			
	48	PLB	36 (75)	54	48 (100)	0 (0)	0 (0)	48 (100)			
Ohtsu (2013) [52]	439	Everolimus	322 (73)	62 (20–86)	439 (100)	25 (6)	0 (0)	118 (27)	438 (99)	EORTC QLQ-C30	0.55
	217	PLB + BSC	161 (74)	62 (20–88)	217 (100)	27 (12)	0 (0)	69 (32)	217 (100)		
Wilke (2014) [54]–Al-Batran (2016) [53]	330	Ramucirumab + PTX	229 (69)	61 (25–83)	NA	0 (0)	0 (0)	66 (20)	264 (80)	EORTC QLQ-C30, EQ 5D	0.82
	335	PTX + PLB	243 (73)	61 (24–84)	NA	0 (0)	0 (0)	71 (21)	264 (79)		

*ACS* Adjusted Checklist Score, *Cap* capecitabine, *CCSQ* Chemotherapy Convenience and Satisfaction Questionnaire, *CI* confidence interval, *Cis* cisplatin, *Doxo* doxorubicin, *DTX* docetaxel, *EORTC* European Organisation for Research and Treatment of Cancer, *Epi* epirubicin, *EQ5D* a standardized instrument for measuring generic health status from the EuroQol Group, *FACT-BRM* Functional Assessment of Cancer Therapy—Biological Response Modifier, *FACT–Fatigue* Functional Assessment of Chronic Illness Therapy–Fatigue, *FACT-GA* Functional Assessment of Cancer Therapy—Gastric Cancer Module, *GEJ*: gastroesophageal junction, *BSC* best supportive care, *HR* hazard ratio, *HU* hydroxyurea, *IMFQ* Interferon-Mediated Fatigue Questionnaire, *Iri* irinotecan, *MMC* mitomycin, *N* sample size, *Ox* oxaliplatin, *PLB* placebo, *Pmab* panitumumab, *PTX* paclitaxel, *Lv* leucovorin, *QLQ-C30* EORTC cancer-specific quality-of-life questionnaire, *QLQ-OES18* EORTC esophageal cancer module, *QLQ-OG25* EORTC esophageal and gastric cancer module, *QLQ-STO22* EORTC gastric cancer module, *SQLI* Spitzer Quality of Life Index, *Tmab* trastuzumab, *VAS-PAIN* visual analogue scale for pain scores, *WHO PS* World Health Organization performance status, *5-FU* fluorouracil

* Karnofsky performance status (0–100) was given instead of the WHO performance status. ** Mean and standard deviation were given instead of median and range

### Checklist

#### Conceptual issues

Only nine studies (24.3%) reported an a priori hypothesis, and two studies (5.4%) stated explicitly that the HRQoL assessment had an exploratory nature. No studies provided a rationale for selecting a specific HRQoL questionnaire (see Table 2). Checklist scores per item per RCT are provided in Online Resource 2 in the ESM.

**Table 2:** Checklist items on the Minimum Standard Checklist for Evaluating HRQoL Outcomes

Checklist items	Mandatory item	Total (%), *N* = 37
Conceptual domain		
A priori hypothesis stated	No	11 (29.7)
Rationale for instrument reported	No	0 (0)
Measurement domain		
Psychometric properties reported	Yes	37 (100)
Cultural validity verified	No	37 (100)
Adequacy of domains covered	No	37 (100)
Methodology domain		
Instrument administration reported	No	8 (21.6)
Baseline compliance reported	Yes	30 (81.1)
Timing of assessments documented	No	36 (97.3)
Missing data documented	Yes	13 (35.1)
Interpretation domain		
Clinical significance addressed	No	15 (40.1)
Presentation of results in general	No	22 (59.5)

#### Measurement issues

All studies used a culturally validated HRQoL questionnaire with previously published psychometric properties and adequate covering of HRQoL domains. The most frequently used questionnaire was the EORTC QLQ-C30 (32 studies, 86.5%) (Table 1). Additional disease-specific questionnaires (e.g., EORTC QLQ-OES18 for esophageal cancer, STO22 for gastric cancer, or OG25 for esophagogastric cancer) were used in addition to the QLQ-C30 (as recommended by the EORTC) in 12 of the 37 studies (32.4%). Five studies (13.5%) used the EQ-5D, four of which were employed in combination with the EORTC QLQ-C30. The Spitzer Quality of Life Index (a proxy-based questionnaire) was used solely in one study (2.7%).

#### Methodology

In eight studies (21.6%), the authors specified by whom and in which clinical setting the HRQoL instrument was administered. Baseline compliance was reported in 30 studies (81.1%), and the timing schedule of the HRQoL assessments was documented in 36 studies (97.3%). Only 13 studies (35.1%) provided reasons for missing data or the number of patients for whom data were missing during the study.

#### HRQoL interpretation

In 15 studies (40.1%), the authors addressed the clinical significance of the HRQoL findings. In 22 studies (59.5%), the authors provided any comments on the HRQoL assessment in their study, regardless of the results.

### Overall quality of HRQoL reporting

**Fig. 2:**
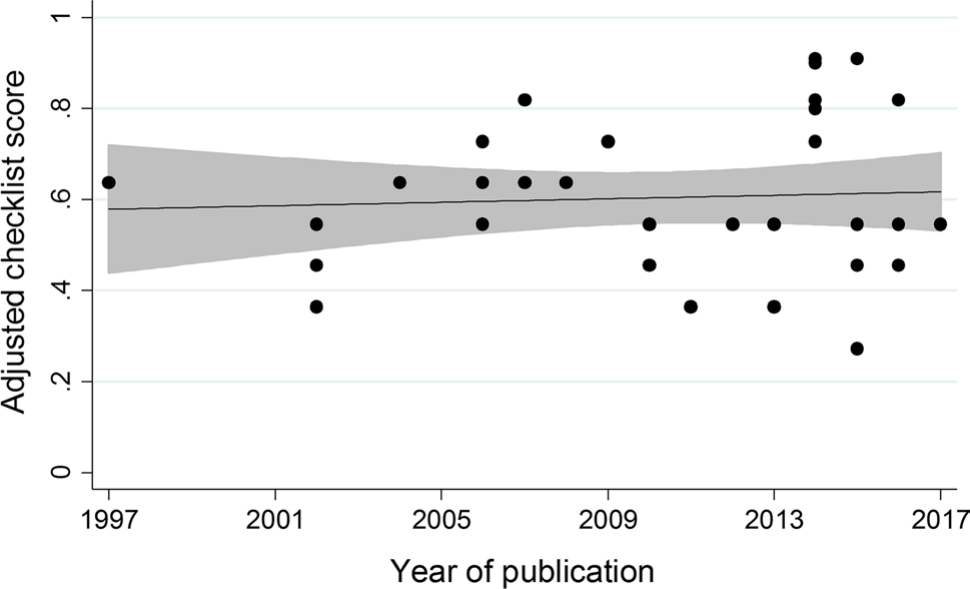
Scatterplot depicting the year of publication versus the adjusted checklist score. The* x-axis* shows the year of publication, and the* y-axis* shows the adjusted checklist score. The* gray area* represents the 95% confidence interval of the mean adjusted checklist score

Among the 37 studies, the quality of 4 studies (10.8%) was classified as ‘very limited,’ that of 24 studies (64.9%) was classified as ‘limited,’ and the quality of 9 studies (24.3%) was classified as ‘probably robust.’ Figure 2 shows that the adjusted checklist scores per study varied over time. A high variability in ACS scores over time and also within publication years can be seen. For all studies, the median quality score was 0.55 and ranged from 0.27 to 0.91. Univariate generalized linear regression analysis showed that the year of publication was not associated with an increased ACS (*β* = 0.004, SE = 0.007, *P* = 0.57). Moreover, there was no difference between the median ACS scores of studies published before [median ACS = 0.55, interquartile range (IQR) = 0.5–0.64, *N* = 20) and after (median ACS = 0.55, IQR = 0.55–0.82, *N* = 17) the publication of the CONSORT-PRO statement, *z* = 1.12, *P* = 0.26. Second-line therapy and the publication of HRQoL results in a separate article were found to be significantly associated with the quality of reporting in the multivariate analysis (Table 3). In addition, post hoc analysis showed similar results when the criterion with the lowest score (’rationale for instrument reported’) was omitted (data not shown).

**Table 3:** Results of the univariate and multivariate generalized linear regression analysis

Univariate analysis			
Covariate	Regression coefficient	Standard error	*P* value
Year of publication (continuous)	0.004	0.007	0.57
Sample size, *N* (continuous)	0.0001	0.0002	0.52
Endpoint (first vs second)	− 0.014	0.102	0.89
Appendix or supplementary material (no vs yes)	0.067	0.116	0.57
Separate article with HRQoL results (no vs yes)	0.353	0.074	< 0.001*
Study differences in HRQoL between or within study arms (no versus yes)	0.184	0.079	0.02*
Treatment line			
First line (reference)			
Second line	0.230	0.123	0.06
Third line	− 0.390	0.096	< 0.001*

Multivariate analysis			
Year of publication (continuous)	− 0.009	0.006	0.16
Separate article with HRQoL results (no vs yes)	0.369	0.088	< 0.001*
Study differences in HRQoL between or within study arms (no versus yes)	0.057	0.077	0.46
Treatment line			
First line (reference)			
Second line	0.243	0.120	0.02*
Third line	− 0.290	0.116	0.07

The dependent variable is the adjusted checklist score

*P values are significant at the 5% level

## Discussion

Although more than half of all the RCTs included in this systematic review were published in the past 5 years, the quality of HRQoL reporting in esophagogastric cancer RCTs involving palliative systemic therapy was limited and did not improve over time. This outcome is independent of the type of endpoint used in the RCT, the usage of supplementary data or appendices in the main publication, and, most importantly, the number of patients in the RCT. The latter indicates that shortcomings in reporting occur in both small and large phase III RCTs. Since larger and otherwise methodologically sound trials are the basis for guideline development and clinical decision-making, we advocate that care should be taken when interpreting HRQoL findings from these trials.

While most included studies report the timing schedule of the HRQoL assessments, describe compliance rates, and use validated questionnaires, the following aspects of HRQoL reporting require improvement: the formulation of a priori hypotheses, a clear description of how the instrument is administered, the interpretation of findings, and the number of missing data as well as how such data are handled (see Table 2). The latter in particular provides valuable information regarding potential bias in HRQoL estimates when there is nonrandom attrition. The importance of reporting missing data is reflected in the checklist, given that it is required before the study can be rated as high quality.

RCTs that presented HRQoL findings in a separate article were significantly more likely to be of better quality than studies that published their HRQoL findings along with the main clinical results. This pattern was also found in the systematic review of Brundage and colleagues [6]. Those authors emphasized that poorer reporting is most likely due to restrictions on manuscript length. Thus, omitting valuable HRQoL data to ensure that the word count is below a particular limit might lead to reporting bias and therefore hamper interpretation and clinical decision-making. Furthermore, publication bias could arise when findings are not significant and/or compliance rates in RCTs are low. Conversely, the publication of HRQoL data separately from the main clinical findings may reduce their clinical impact. For these reasons, one could consider reporting HRQoL findings in an extensive appendix or supplementary dataset along with the main article, so that valuable information regarding both clinical and HRQoL outcomes can be presented within one publication.

As observed previously, we found substantial variability in the quality of HRQoL reporting [4, 6, 7, 57]. The Consolidated Standards of Reporting Trials Regarding Patient-Reported Outcomes (CONSORT-PRO) statement provides detailed information on how to accurately and transparently report HRQoL in RCTs, and is endorsed by prominent journals. The current systematic review suggests that the CONSORT-PRO statement may not have had a significant impact on the reporting of HRQoL findings in esophagogastric cancer yet [10, 57].

Our study has some limitations. First, the Minimum Standard Checklist for Evaluating HRQoL Outcomes in Cancer Clinical Trials was published in 2003 and might not be as extensive as those published later, such as the CONSORT PRO or the ISOQOL-recommended PRO reporting standards (both published in 2013) [10, 58]. The advantage of the checklist used is the predefined scoring system. In addition, the checklist includes the majority of the essential items of the latter published statements and recommendations based on expert consensus by CONSORT PRO and ISOQOL, respectively. The checklist is based on a minimum set of criteria, whereas the CONSORT-PRO or the ISOQOL-recommended PRO reporting standards elaborate more extensively on different aspects of HRQoL assessments. Extensive tools may be more sensitive to change, which means that the results in the current study might be an underestimation of the true change that occurred over time [7].

Second, the search strategy was limited to reports in English. Consequently, we might have failed to include RCT reports published in other languages—thus limiting our international scope. However, since the major phase II/III trials are published in English, we believe the risk of language bias to be low.

Third, RCTs scored particularly poorly on the item ‘rationale for the instrument used.’ The validated EORTC QLQ-C30 questionnaire is most frequently used in esophagogastric cancer, and this can be regarded as the ‘standard’ HRQoL instrument in esophagogastric cancer RCTs. Therefore, devaluing a RCT for not stating a rationale for the instrument used may be an excessively strict approach, as the EORTC questionnaire is consistently applied in order to permit fair comparisons between trials. However, post hoc analysis showed that the results were not different when the criterion ‘rationale for instrument reported’ was omitted. It should be emphasized that when authors use a newly developed or less frequently applied questionnaire, they should state the rationale.

Finally, one might dispute the interpretation of the outcome (ACS) as an interval scale. We adhered to the general practice of analyzing percentages or values between 0 and 1 to two decimal places using parametric statistical techniques.

To improve the quality of HRQoL reporting in future RCTs, we recommend that researchers and clinicians should involve a HRQoL expert in the trial design, execution, analysis, and reporting phases. When the word count is restricted by journals, an extensive appendix or supplementary dataset can be of value. In addition, we would like to affirm the comment by Brundage et al. [6] that researchers and clinicians in advisory positions can stimulate the acceptance of patient-reported outcome reporting standards—such as the CONSORT PRO—by involving editors, reviewers, and related stakeholders.

## Conclusion

Although the number of RCTs on palliative systemic therapy for advanced esophagogastric cancer that include an HRQoL endpoint has increased, the quality of HRQoL reporting is highly variable, limited, and did not improve over time. This systematic review highlights the gaps in the current quality of HRQoL reporting in esophagogastric cancer RCTs. The formulation of a priori hypotheses, a clear description of how the instrument is administered, the number of missing data and how those data are handled, and the interpretation of findings are areas for improvement. We recommend that HRQoL should be extensively described in supplementary appendices if good HRQoL reporting is restricted by the word limit of the manuscript. As results from RCTs are crucial to daily practice, reliable and adequate reporting of HRQoL outcomes from RCTs is needed to facilitate clinical decision-making.

## Electronic supplementary material

Below is the link to the electronic supplementary material.

Supplementary material 1 (PDF 21 kb)

Supplementary material 2 (PDF 148 kb)
