# Supplementary material for: Reporting of health-related quality of life in randomized controlled trials involving palliative systemic therapy for esophagogastric cancer: a systematic review
Source: Gastric Cancer. 2018 Jan 29;21(2):183–95. doi: 10.1007/s10120-018-0792-3 (PMC5846827; doi:10.1007/s10120-018-0792-3)
Supplement: Supplementary file 1 — Supplementary material 1 (PDF 21 kb) [file 10120_2018_792_MOESM1_ESM.pdf]

Gastric Cancer

Reporting of health-related quality of life in randomized controlled trials involving palliative systemic therapy for esophagogastric cancer: A systematic review.

Emil ter Veer [1]*, Jessy Joy van Kleef [1]*, Mirjam A.G. Sprangers [2], Nadia Haj Mohammad [3], Martijn G.H. van Oijen [1] and Hanneke W.M. van Laarhoven [1] .

[1] Cancer Center Amsterdam, Department of Medical Oncology, Academic Medical Center, University of Amsterdam, Meibergdreef 9, 1105 AZ, Amsterdam, the Netherlands.
[2] Academic Medical Center, University of Amsterdam, Department of Medical Psychology, Amsterdam Public Health research institute, Meibergdreef 9, 1105 AZ, Amsterdam, the Netherlands.
[3] Department of Medical Oncology, University Medical Center Utrecht, Heidelberglaan 100, 3584 CX Utrecht, the Netherlands.

* contributed equally

Address for correspondence:
E. ter Veer
Cancer Center Amsterdam, Department of Medical Oncology, Academic Medical Center
Meibergdreef 9; F4-224
1105 AZ Amsterdam
tel: +31 (0)20-5665955; e.terveer@amc.uva.nl

Cochrane Central Register of Controlled Trials (CENTRAL)

ID	Search
#1	MeSH descriptor: [Esophageal Neoplasms] explode all trees
#2	MeSH descriptor: [Stomach Neoplasms] explode all trees
#3	((esophag* or oesophag* or stomach or gastric or gastroesophag* or gastrooesophag*) and (neoplas* or cancer* or carcino* or adenocarcino* or tumor or tumors or tumour or tumours or malig*)):ti,ab,kw
#4	#1 or #2 or #3
#5	MeSH descriptor: [Palliative Care] explode all trees
#6	MeSH descriptor: [Neoplasm Metastasis] explode all trees
#7	(palliat* or advanced or metasta* or irresect* or unresect* or un-resect* or non-resect* or nonresect* or inopera* or non-opera* or nonopera* or unopera*):ti,ab,kw
#8	#5 or #6 or #7
#9	MeSH descriptor: [Drug Therapy, Combination] explode all trees
#10	MeSH descriptor: [Drug Combinations] explode all trees
#11	MeSH descriptor: [Antineoplastic Agents] explode all trees
#12	MeSH descriptor: [Anthracyclines] explode all trees
#13	MeSH descriptor: [Leucovorin] explode all trees
#14	MeSH descriptor: [Organoplatinum Compounds] explode all trees
#15	MeSH descriptor: [Oxonic Acid] explode all trees
#16	MeSH descriptor: [Taxoids] explode all trees
#17	(chemotherap* or polytherap* or polychemotherap* or combination* or two-agent* or two-drug* or double-drug* or doublet* or three-agent* or three-drug* or triple* or multi-agent or multi-drug or active agent* or antineoplastic* or anti-neoplastic* or anticancer* or anti-cancer* or antitumor* or anti-tumor* or antitumour* or anti-tumour* or anthracyclin* or capecitabine or carboplatin* or cisplatin* or docetaxel or doxorubicin* or epirubicin* or fluoropyrimidine* or fluorouracil or 5-FU or folinic acid or irinotecan or leucovorin* or mitomycin* or organoplatin* or oteracil or oxaliplatin* or oxonic acid or paclitaxel or platin* or S-1 or taxane* or tegafur):ti,ab,kw
#18	#9 or #10 or #11 or #12 or #13 or #14 or #15 or #16 or #17
#19	#4 and #8 and #18 in Trials

EMBASE via Ovid

1. esophagus tumor/ or exp esophagus cancer/
2. stomach tumor/ or exp stomach cancer/
3. ((esophag* or oesophag* or stomach or gastric or gastroesophag* or gastrooesophag*) adj5 (neoplas* or cancer* or carcino* or adenocarcino* or tumor or tumors or tumour or tumours or malig*)).ti,ab.
4. or/1-3
5. exp cancer palliative therapy/
6. exp metastasis/
7. advanced cancer/
8. inoperable cancer/
9. (palliat* or advanced or metasta* or irresect* or unresect* or un-resect* or non-resect* or nonresect* or inopera* or non-opera* or nonopera* or unopera*).ti,ab.
10. or/5-9
11. exp drug combination/
12. exp antineoplastic agent/
13. folinic acid/

14. platinum complex/
15. oteracil/
16. taxane derivative/
17. taxoid/
18. (chemotherap* or polytherap* or polychemotherap* or combination* or two-agent* or two-drug* or double-drug* or doublet* or three-agent* or three-drug* or triple* or multi-agent or multi-drug or active agent* or antineoplastic* or anti-neoplastic* or anticancer* or anti-cancer* or antitumor* or anti-tumor* or antitumour* or anti-tumour* or anthracyclin* or capecitabine or carboplatin* or cisplatin* or docetaxel or doxorubicin* or epirubicin* or fluoropyrimidine* or fluorouracil or 5-FU or folinic acid or irinotecan or leucovorin* or mitomycin* or organoplatin* or oteracil or oxaliplatin* or oxonic acid or paclitaxel or platin* or S-1 or taxane* or tegafur).ti,ab.
19. or/11-18
20. exp controlled clinical trial/ or randomized.ti,ab. or randomised.ti,ab. or placebo.ti,ab. or randomly.ti,ab. or trial.ti.
21. 4 and 10 and 19 and 20
22. limit 21 to (dutch or english)
23. limit 22 to (conference abstract or conference paper or "conference review" or conference proceeding)
24. 22 not 23

Medline via Pubmed

("Esophageal Neoplasms"[Mesh] OR "Stomach Neoplasms"[Mesh] OR ((esophag*[tiab] OR oesophag*[tiab] OR stomach[tiab] OR gastric[tiab] OR gastroesophag*[tiab] OR gastrooesophag*[tiab]) AND (neoplas*[tiab] OR cancer*[tiab] OR carcino*[tiab] OR adenocarcino*[tiab] OR tumor[tiab] OR tumors[tiab] OR tumour[tiab] OR tumours[tiab] OR malig*[tiab])))

AND

("Palliative Care"[Mesh] OR "Neoplasm Metastasis"[Mesh] OR palliat*[tiab] OR advanced[tiab] OR metasta*[tiab] OR irresect*[tiab] OR unresect*[tiab] OR un-resect*[tiab] OR non-resect*[tiab] OR nonresect*[tiab] OR inopera*[tiab] OR non-opera*[tiab] OR nonopera*[tiab] OR unopera*[tiab])

AND

("Drug Therapy, Combination"[Mesh] OR "Drug Combinations"[Mesh] OR "Antineoplastic Agents"[Mesh] OR "Antineoplastic Agents"[Pharmacological Action] OR "Anthracyclines"[Mesh] OR "Leucovorin"[Mesh] OR "Organoplatinum Compounds"[Mesh] OR "Oxonic Acid"[Mesh] OR "Taxoids"[Mesh] OR chemotherap*[tiab] OR polytherap*[tiab] OR polychemotherap*[tiab] OR combination*[tiab] OR two-agent*[tiab] OR two-drug*[tiab] OR double-drug*[tiab] OR doublet*[tiab] OR three-agent*[tiab] OR three-drug*[tiab] OR triple*[tiab] OR multi-agent[tiab] OR multi-drug[tiab] OR active agent*[tiab] OR antineoplastic*[tiab] OR anti-neoplastic*[tiab] OR anticancer*[tiab] OR anti-cancer*[tiab] OR antitumor*[tiab] OR anti-tumor*[tiab] OR antitumour*[tiab] OR anti-tumour*[tiab] OR anthracyclin*[tiab] OR capecitabine[tiab] OR carboplatin*[tiab] OR cisplatin*[tiab] OR docetaxel[tiab] OR doxorubicin*[tiab] OR epirubicin*[tiab] OR fluoropyrimidine*[tiab] OR fluorouracil[tiab] OR 5-FU[tiab] OR folinic acid[tiab] OR irinotecan[tiab] OR leucovorin*[tiab] OR mitomycin*[tiab] OR organoplatin*[tiab] OR oteracil[tiab] OR oxaliplatin*[tiab] OR oxonic acid[tiab] OR paclitaxel[tiab] OR platin*[tiab] OR S-1[tiab] OR taxane*[tiab] OR tegafur[tiab]

AND

(randomized controlled trial[pt] OR controlled clinical trial[pt] OR randomized[tiab] OR randomised[tiab] OR placebo[tiab] OR clinical trials as topic[mesh:noexp] OR randomly[tiab] OR trial[ti]) AND (english[la] OR dutch[la])

Conference search: American Society of Clinical Oncology
Searching journal content for gastric (all words) in title or abstract and random* OR advance* OR metasta* (all words) in full text, from earliest publication date through February 2017

Conference search: European Society of Medical Oncology
Searching journal content for gastric (all words) in title or abstract and random* OR advance* OR metasta* (all words) in full text, from earliest publication date through February 2017
