# Supplementary material for: Reporting of health-related quality of life in randomized controlled trials involving palliative systemic therapy for esophagogastric cancer: a systematic review
Source: Gastric Cancer. 2018 Jan 29;21(2):183–95. doi: 10.1007/s10120-018-0792-3 (PMC5846827; doi:10.1007/s10120-018-0792-3)
Supplement: Supplementary file 2 — Supplementary material 2 (PDF 148 kb) [file 10120_2018_792_MOESM2_ESM.pdf]

# Gastric Cancer

# Reporting of health-related quality of life in randomized controlled trials involving palliative systemic therapy for esophagogastric cancer: A systematic review.

Emil ter Veer [1]*, Jessy Joy van Kleef [1]*, Mirjam A.G. Sprangers [2], Nadia Haj Mohammad [3], Martijn G.H. van Oijen [1] and Hanneke W.M. van Laarhoven [1] .

[1] Cancer Center Amsterdam, Department of Medical Oncology, Academic Medical Center, University of Amsterdam, Meibergdreef 9, 1105 AZ, Amsterdam, the Netherlands.
[2] Academic Medical Center, University of Amsterdam, Department of Medical Psychology, Amsterdam Public Health research institute, Meibergdreef 9, 1105 AZ, Amsterdam, the Netherlands.
[3] Department of Medical Oncology, University Medical Center Utrecht, Heidelberglaan 100, 3584 CX Utrecht, the Netherlands.

* contributed equally

**Address for correspondence:**
E. ter Veer
Cancer Center Amsterdam, Department of Medical Oncology, Academic Medical Center
Meibergdreef 9; F4-224
1105 AZ Amsterdam
tel:  +31 (0)20-5665955; e.terveer@amc.uva.nl

**Online Resource 2: Scores of individual randomized controlled trials on the 'minimum standard checklist for evaluating HRQoL outcomes in cancer clinical trials'.**

| **Study** | **Crit 1** | **Crit 2** | **Crit 3** | **Crit 4** | **Crit 5** | **Crit 6** | **Crit 7** | **Crit 8** | **Crit 9** | **Crit 10** | **Crit 11** | **Total Score/ Total items** | **Number Mandatory Items Present** | **ACS** |
|---|---|---|---|---|---|---|---|---|---|---|---|---|---|---|
| Ajani 2010 - Bodoky 2015 | + | - | + | + | + | + | + | + | + | + | + | 10/11 | 3 | 0.91 |
| Al-Batran 2013 - Kripp 2014 | + | - | + | + | + | - | + | + | - | + | + | 8/11 | 2 | 0.73 |
| Bang 2010 - Satoh 2014 | + | - | + | + | + | + | + | + | - | + | + | 9/11 | 2 | 0.82 |
| Bouche 2004 | - | - | + | + | + | - | + | + | + | - | + | 7/11 | 3 | 0.64 |
| Bramhall 2002 | - | - | + | + | + | - | - | + | - | - | - | 4/11 | 1 | 0.36 |
| Dank 2008 - Curran 2009 | + | - | + | + | + | - | + | + | + | - | + | 8/11 | 3 | 0.73 |
| Duffour 2006 | - | - | + | + | + | - | + | + | - | - | + | 6/11 | 2 | 0.55 |
| Dutton 2014 | * | - | + | + | + | + | + | + | + | + | + | 9/10 | 3 | 0.90 |

| | | | | | | | | | | | | | | |
|---|---|---|---|---|---|---|---|---|---|---|---|---|---|---|
| Ford 2014 | + | - | + | + | + | + | + | + | + | + | + | 10/11 | 3 | 0.91 |
| Fuchs 2014 | - | - | + | + | + | - | + | + | + | + | + | 8/11 | 3 | 0.73 |
| Glimelius 1997 | - | - | + | + | + | - | + | + | + | - | + | 7/11 | 3 | 0.64 |
| Gubanski 2010 - Gubanski 2014 | * | - | + | + | + | - | + | + | + | + | + | 8/10 | 3 | 0.80 |
| Guimbaud 2014 - Nuami 2015 | - | - | + | + | + | - | - | + | + | - | + | 6/11 | 2 | 0.55 |
| Hall 2017 | - | - | + | + | + | + | + | + | - | - | - | 6/11 | 2 | 0.55 |
| Hecht 2015 | - | - | + | + | + | - | - | - | - | - | - | 3/11 | 1 | 0.27 |
| Hwang 2017 | - | - | + | + | + | - | + | + | - | + | - | 6/11 | 2 | 0.55 |
| Kim 2012 | + | - | + | + | + | - | + | + | - | - | - | 6/11 | 2 | 0.55 |
| Lee 2016 | - | - | + | + | + | - | - | + | - | + | - | 5/11 | 1 | 0.45 |
| Li 2013 | - | - | + | + | + | - | - | + | - | - | - | 4/11 | 1 | 0.36 |
| Li 2016 | - | - | + | + | + | - | + | + | - | - | - | 5/11 | 2 | 0.45 |

| | | | | | | | | | | | | | | |
|---|---|---|---|---|---|---|---|---|---|---|---|---|---|---|
| Ohtsu 2011 | - | - | + | + | + | - | - | + | - | - | - | 4/11 | 1 | 0.36 |
| Ohtsu 2013 | + | - | + | + | + | - | + | + | - | - | - | 6/11 | 2 | 0.55 |
| Park 2006 | - | - | + | + | + | - | + | + | - | + | + | 7/11 | 2 | 0.64 |
| Park 2008 | - | - | + | + | + | + | + | + | - | - | + | 7/11 | 2 | 0.64 |
| Rao 2010 | - | - | + | + | + | - | + | + | - | - | - | 5/11 | 2 | 0.45 |
| Ross 2002 | - | - | + | + | + | - | + | + | - | - | + | 6/11 | 2 | 0.55 |
| Roth 2007 | - | - | + | + | + | - | + | + | - | + | + | 7/11 | 2 | 0.64 |
| Ryu 2015 | - | - | + | + | + | - | + | + | - | - | - | 5/11 | 2 | 0.45 |
| Sadighi 2006 | - | - | + | + | + | - | + | + | + | + | + | 8/11 | 3 | 0.73 |
| Tebbutt 2002 | - | - | + | + | + | + | - | + | - | - | - | 5/11 | 1 | 0.45 |
| Tebbutt 2016 | - | - | + | + | + | - | + | + | - | + | - | 6/11 | 2 | 0.55 |
| Tebbutt2010 | - | - | + | + | + | - | + | + | - | + | - | 6/11 | 2 | 0.55 |
| Van Cutsem 2006 - Ajani 2007 | + | - | + | + | + | + | + | + | + | - | + | 9/11 | 3 | 0.82 |

| | | | | | | | | | | | | | | |
|---|---|---|---|---|---|---|---|---|---|---|---|---|---|---|
| Wadler 2002 | - | - | + | + | + | - | + | + | - | - | + | 6/11 | 2 | 0.55 |
| Webb 1997 | - | - | + | + | + | - | + | + | + | - | + | 7/11 | 3 | 0.64 |
| Wilke 2014 - Al-Batran 2016 | + | - | + | + | + | - | + | + | + | + | + | 9/11 | 3 | 0.82 |
| Yoshino 2016 | - | - | + | + | + | - | + | + | - | - | + | 6/11 | 2 | 0.55 |

Abbreviations: Crit: Criterion, ACS: adjusted checklist score. *HRQoL analyses had an exploratory nature. Mandatory items are criteria 3,7 and 9.

A plus sign indicates that the criterion of interest is described in the study report. A minus sign indicates that the criterion of interest is not (fully) described in the study report.

Criterion 1: A priori hypothesis stated, Criterion 2: Rationale for instrument reported, Criterion 3: Psychometric properties reported, Criterion 4: Cultural validity verified, Criterion 5: Adequacy of domains covered, Criterion 6: Instrument administration reported, Criterion 7: Baseline compliance reported, Criterion 8: Timing of assessments documented, Criterion 9: Missing data documented, Criterion 10: Clinical significance addressed, Criterion 11: Presentation of results in general.
